# Α Virtual Reality App for Physical and Cognitive Training of Older People With Mild Cognitive Impairment: Mixed Methods Feasibility Study

**DOI:** 10.2196/24170

**Published:** 2021-03-24

**Authors:** Mary Hassandra, Evangelos Galanis, Antonis Hatzigeorgiadis, Marios Goudas, Christos Mouzakidis, Eleni Maria Karathanasi, Niki Petridou, Magda Tsolaki, Paul Zikas, Giannis Evangelou, George Papagiannakis, George Bellis, Christos Kokkotis, Spyridon Rafail Panagiotopoulos, Giannis Giakas, Yannis Theodorakis

**Affiliations:** 1 School of Physical Education, Sport Science and Dietetics Department of Physical Education and Sport Science University of Thessaly Trikala Greece; 2 Greek Association of Alzheimer’s Disease & Related Disorders Alzheimer Hellas Thessaloniki, Makedonia Greece; 3 1st Department of Neurology, School of Medicine, Faculty of Health Sciences Aristotle University of Thessaloniki Thessaloniki, Makedonia Greece; 4 ORamaVR S.A. Science and Technology Park of Crete Heraklion, Crete Greece; 5 Institute of Computer Science Foundation for Research and Technology – Hellas (FORTH) University of Crete Heraklion, Crete Greece; 6 Biomechanical Solutions Engineering (BME) Karditsa Greece

**Keywords:** virtual reality, elderly, mild cognitive impairment, combined physical and cognitive function, dual task

## Abstract

**Background:**

Therapeutic virtual reality (VR) has emerged as an effective treatment modality for cognitive and physical training in people with mild cognitive impairment (MCI). However, to replace existing nonpharmaceutical treatment training protocols, VR platforms need significant improvement if they are to appeal to older people with symptoms of cognitive decline and meet their specific needs.

**Objective:**

This study aims to design and test the acceptability, usability, and tolerability of an immersive VR platform that allows older people with MCI symptoms to simultaneously practice physical and cognitive skills on a dual task.

**Methods:**

On the basis of interviews with 20 older people with MCI symptoms (15 females; mean age 76.25, SD 5.03 years) and inputs from their health care providers (formative study VR1), an interdisciplinary group of experts developed a VR system called VRADA (VR Exercise App for Dementia and Alzheimer’s Patients). Using an identical training protocol, the VRADA system was first tested with a group of 30 university students (16 females; mean age 20.86, SD 1.17 years) and then with 27 older people (19 females; mean age 73.22, SD 9.26 years) who had been diagnosed with MCI (feasibility studies VR2a and VR2b). Those in the latter group attended two Hellenic Association Day Care Centers for Alzheimer’s Disease and Related Disorders. Participants in both groups were asked to perform a dual task training protocol that combined physical and cognitive exercises in two different training conditions. In condition A, participants performed a cycling task in a lab environment while being asked by the researcher to perform oral math calculations (single-digit additions and subtractions). In condition B, participants performed a cycling task in the virtual environment while performing calculations that appeared within the VR app. Participants in both groups were assessed in the same way; this included questionnaires and semistructured interviews immediately after the experiment to capture perceptions of acceptability, usability, and tolerability, and to determine which of the two training conditions each participant preferred.

**Results:**

Participants in both groups showed a significant preference for the VR condition (students: mean 0.66, SD 0.41, *t*_29_=8.74, *P*<.001; patients with MCI: mean 0.72, SD 0.51, *t*_26_=7.36, *P*<.001), as well as high acceptance scores for intended future use, attitude toward VR training, and enjoyment. System usability scale scores (82.66 for the students and 77.96 for the older group) were well above the acceptability threshold (75/100). The perceived adverse effects were minimal, indicating a satisfactory tolerability.

**Conclusions:**

The findings suggest that VRADA is an acceptable, usable, and tolerable system for physical and cognitive training of older people with MCI and university students. Randomized controlled trial studies are needed to assess the efﬁcacy of VRADA as a tool to promote physical and cognitive health in patients with MCI.

## Introduction

### Background

With an aging population across developed countries, ensuring an independent and healthy lifestyle for older people has become a key social issue and a global public health priority [1]. In this regard, the World Health Organization (WHO) has called for more research to identify ways to support the needs of people living with dementia. According to the WHO, “dementia is a syndrome, of a chronic or progressive nature, in which there is deterioration in cognitive function beyond what might be expected from normal aging.” The condition affects memory, thinking, orientation, comprehension, calculation, learning capacity, language, and judgment. Impaired cognitive function is commonly accompanied or preceded by deteriorating motivation, emotional control, or social behavior. At any given time, an estimated 5% to 8% of people aged 60 years and above have dementia; the global total is projected to reach 82 million by 2030 and 152 million by 2050.

Alzheimer disease (AD) is the most common form of dementia and accounts for 60% to 70% of cases [2]. This is often preceded by a predementia stage known as mild cognitive impairment (MCI), which is an intermediate state between normal aging and dementia involving memory deﬁcits [3] and difficulties with language, thinking, and judgment beyond normal age-related changes that may not be obvious in everyday activities. MCI may be amnesic, nonamnesic, or single- or multiple-domain. The amnesic type includes memory loss and is regarded as a transition stage between normal aging and AD [4]. Studies suggest that 5%-20% of individuals with MCI will develop dementia each year [5,6]. Although MCI does not meet the criteria for dementia, it affects healthy aging and indicates a possible need for medical care and treatment. As all forms of degenerative dementia are incurable, treatment focuses primarily on slowing its progression and managing symptoms, typically through a combination of medication and lifestyle changes [7,8].

### Physical and Cognitive Training

Among nonpharmacological treatments for MCI, combined physical and cognitive training programs are now gaining wider acceptance as part of the standard treatment for people with symptoms of dementia [5,9-11]. Research suggests that physical and cognitive development are interdependent and closely related [12-17], probably because neurogenesis continues even in older adulthood [18,19], and physical exercise is a key dose-related facilitator of neurogenesis [20,21]. New experiences provided by systematic and intense exercise promote alterations in the brain that can contribute to cognitive rehabilitation. Recent studies have reported improved cognitive performance following combined physical and cognitive activities compared with either one alone [22]. This suggests that simultaneous execution of cognitive and motor tasks may yield the greatest improvements in cognitive function [23]. This *dual task* testing requires significant cognitive control of attentional and executive functions and helps to identify patients with MCI who are at high risk of developing dementia [24]. Dual task training has been tested in dementia intervention studies [23,25,26] and has shown promise in patients with neurological disorders (including MCI) as a means of improving balance [27], gait, and cognitive ability [28]. Preliminary evidence also suggests that direct and indirect interventions targeting cognitive-motor interference can help older people with neurodegenerative diseases [29].

Early diagnosis of MCI, including subtype and stage, facilitates earlier treatment and care that can minimize the onset of neurodegeneration, optimize cognitive and physical functioning, and improve quality of life. Emerging technologies offer novel options for research and practice; among these, virtual reality (VR) is a valuable addition as a safe and controlled environment for user interaction and monitoring of physical activity and cognitive task performance. Manipulation of experimental parameters in VR apps has great potential for new forms of dementia intervention and treatment [30-32].

### VR for Combined Physical and Cognitive Training

VR has shown potential as a tool for assessing and training patients diagnosed with dementia and MCI [33,34]. According to recent reviews, VR-based training interventions can be used to improve well-being, cognition, and physical fitness in people with MCI [30,35,36]. Among recent VR systems combining physical training and cognitive training [37,38], the system by Mrakic-Sposta et al [37] involved sequential tasks of riding a bike in a park and avoiding cars while crossing the road to a supermarket. When the system was tested on 10 older patients with MCI and a control group, the results indicated some improvement in cognitive functions, including visual-constructive, visuo-spatial attention, executive, and memory functions, as well as verbal ﬂuency. However, none of the changes was statistically significant [37]. In a more recent randomized controlled trial (RCT) [38], a 12-week VR-based physical and cognitive training program led to signiﬁcant improvements in dual task gait performance among older people with MCI. The physical training elements included Tai Chi, resistance and aerobic exercises, and functional tasks such as window cleaning and goldfish scooping. The cognitive elements included VR games such as buying tickets from vending machines, finding items in a virtual store, and preparing meals as a kitchen chef. Both of these dual task systems for patients with MCI [37,38] were sequential rather than simultaneous. Although closer to everyday situations, these programs also involved multiple cognitive tasks, and some were difficult to compare with standard treatment. In addition, the equipment is expensive and complicated and requires trained specialists to make decisions about system settings and special guidance for participants to ensure efficient training.

On the basis of earlier studies, we designed a simultaneous dual task VR system for physical and cognitive training to support people with MCI. Drawing on clinical ﬁndings regarding MCI rehabilitation and new VR technology, we adopted a person-centered approach to develop a user-friendly, immersive VR training system called VRADA (VR Exercise App for Dementia and Alzheimer’s Patients). The program content was based on previously tested combined protocols for physical and cognitive therapy [39]. In addition, the VRADA exercises allow for future comparisons with standard care and training protocols.

### Design of VR Training Environments

Immersive VR enables researchers to create realistic environments while maintaining a high level of experimental control of essential elements, such as visual and audio feedback and virtual characters. The experience of a virtual body in an immersive virtual environment is similar to the sensations of the biological body [40], and there is a profound link between embodiment and learning [41]. The concept of *presence* refers to the phenomenon of behaving and feeling as if we are actually in the virtual world. This powerful sensation is unique to VR and cannot be created in any other medium. Most people find this magical; unlike *immersion*, where the user is simply surrounded by digital screens, VR enriches immersion and embodiment in a realistic multisensory environment.

Research on the learning impact of embodiment in virtual multimodal environments [42] shows that these settings can facilitate skill transfer when deployed realistically. As embodied navigation and memory are closely linked [43], virtual promenades can compensate for reduced spontaneous motion in older people [44]. In general, embodiment is valuable in these training scenarios because the motivation to experience actions that the situation demands links the user psychologically to the virtual world. VR technology facilitates mixed-ability learning and knowledge transfer and helps participants to interact and collaborate fruitfully through different modalities, and there is evidence that immersive virtual environments enhance training in motor and spatial activities [45,46].

### Person-Centered Approach

According to the Alzheimer Society, a person-centered approach is strongly encouraged in dementia care settings, tailoring care to the individual’s interests, abilities, history, and personality [47]. This umbrella term is used in different disciplines to describe a model that promotes personal autonomy or self-determination within one’s environment. On the basis of self-determination theory, this approach emphasizes the importance of understanding the motivation that drives a person’s behavior and the extent of that motivation. The distinction between autonomous and controlled forms of motivation is central to the theory [48,49].

Neurocognitive disorders are commonly associated with symptoms of apathy and are expressed as low motivation and interest in daily activities, which are known to increase the risk of progression from MCI to AD [50,51]. Therefore, encouragement and motivation are important components of every training program. To address the issue of low motivation to exercise and adherence to training among patients with MCI, we incorporated the following motivational techniques [52] in the VRADA training system. These are derived mainly from self-determination theory [53,54].

- Goal setting: choices for exercise duration when starting each training session
- Feedback on behavior: informative or evaluative feedback at the end of each session on training performance (total distance, cycling time, and number of correct answers on cognitive exercises)
- Task crafting (enjoyment): choice of music to enjoy during training
- Self-monitoring of behavior: screen displays indicating time, speed, and distance for monitoring performance while exercising

The VRADA system design is based on human-centered design, which is a systematic method for developing usable products, systems, or services by focusing explicitly on the intended user [55]. The goal is to maximize relevance and usability, which are defined as the extent to which specified users can use a product to achieve specified goals effectively, efficiently, and with satisfaction in a specified context of use. There is evidence that involving users in the design and development of a new system will improve the system’s quality by ensuring a more accurate assessment of user requirements and a higher level of user acceptance [56]. This study followed these design principles, strategies, and recommended best practices for formative and feasibility studies [57].

### Objectives

This study has 2 main objectives: (1) to describe the formative study of the VRADA system, focusing on how content was developed by working with patients and health care providers and applying the principles of human-centered design and continuous testing (VR1) and (2) to report 2 studies assessing the acceptability, usability, and tolerability of the VRADA system among a sample of university students and a sample of older people with MCI (VR2a and VR2b).

## Methods

### Formative Study VR1

In designing the VRADA system, we adopted a human-centered approach by involving patients (as future users) from the early stages, conducting individual interviews to learn about their relevant needs, experiences, attitudes, beliefs, preferences, aspirations, and expectations, and documenting diverse opinions by gender, education level, and cognitive disorder. From the outset of project planning, we also involved health professionals from the Alzheimer Hellas Day Centers, which are responsible for patients’ physical and cognitive training in order to consult with them during the design, application, and evaluation phases of the VRADA project.

#### Study Aim

The aim of this study is to collect information about patients’ training experiences, preferences, and expectations to develop a user-centered training system.

#### Participants and Setting

Patients were recruited for interviews at 2 Hellenic Alzheimer Association Day Centers in Thessaloniki in August 2018. Older people visit these centers voluntarily for neuropsychological assessments, neurological examinations, and case management. A total of 20 patients volunteered to participate in the study (15 women and 5 men; mean age 76.25, SD 5.03 years; age range 69-84 years). In total, 16 had been diagnosed with MCI and 4 with subjective cognitive decline; most patients were retired (mean years of education 11.35, SD 5.76; range 5-18 years).

#### Assessments

The 2 trained psychologists who worked at the 2-day care centers conducted the interviews using an open-ended questionnaire as a guide. The interview topics were type, frequency, and duration of exercise sessions the interviewee attended at the day care center and whether they engaged in any additional physical activity elsewhere; preferred outdoor exercise environment (scenery, season, time of day, social environment, and music); feedback options while exercising (speed, time, distance, and heart rate); standing bike preferences (familiarity with biking and sitting bike vs upright bike); and cognitive exercises they would most enjoy while biking (memory, attention, and problem solving).

#### Procedure and Data Analysis

Two interviewers invited older people to participate voluntarily in the study. The first 20 who accepted then signed informed consent forms, and all participants agreed to the use of a voice recorder. Interviews lasted for 30 minutes to 1 hour and were subsequently transcribed verbatim for thematic analysis of each topic or question. All answers were organized in tabular format, and similar answer frequencies were calculated.

### Feasibility Studies VR2a and VR2b

#### Study Aim

In the 2 identical test studies with university students (VR2a) and end users (VR2b), we focused on the acceptability, usability, and tolerability of using the VRADA system as compared with standard care physical and cognitive training.

#### Participants

A total of 30 undergraduate students (14 males and 16 females) from a physical education and sports science department participated in study VR2a. The mean age of the participants was 20.86 years (SD 1.17). In study VR2b, 27 participants (8 males and 19 females) were recruited from 2-day care centers run by the Greek Association of Alzheimer's Disease and Related Disorders - Alzheimer Hellas. The latter group comprised patients who had been diagnosed with MCI according to the Petersen criteria [58] and were at stage 3 of the disease according to the Global Deterioration Scale [59], with a Clinical Dementia Rating [60] score of 0.5 and exhibiting subjective cognitive decline [61]. They ranged in age from 59 to 85 years (mean age 73.22, SD 9.26 years). Demographic details (educational level, exercise habits, and use of technology) are presented in Multimedia Appendix 1.

#### Apparatus

A cycle-ergometer (stationary seated bike type; Toorx, Chrono Line, BRX R 300) was identified as the optimal choice for the exercise apparatus, as it has been proven to reduce user fall risk and facilitate precise control of training conditions. It also meets the requirements of Bluetooth connectivity capability.

#### Application Description

**Figure 1:**
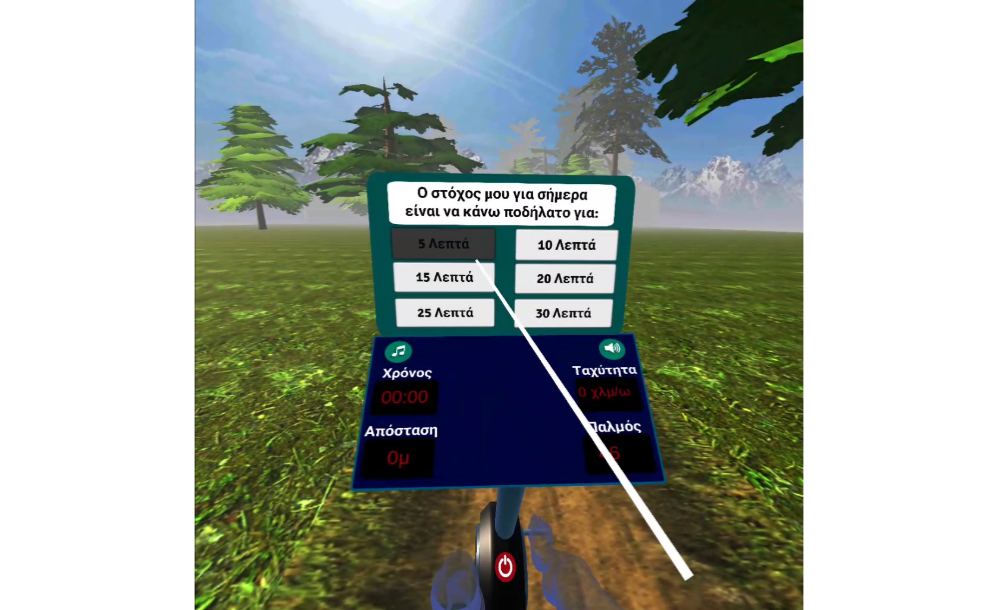
User selects the daily goal for the duration of the training session.

This section details the functionalities and phases of the VR apps used (a demo video is available in Multimedia Appendix 2). On first running the VRADA app, the user must select the number of minutes they aim to cycle within the virtual environment. As a selection mechanism, we implemented a raycast from the VR controller, allowing the user to select an answer by pointing the ray at the button and pressing the trigger button on the controller (Figure 1).

**Figure 2:**
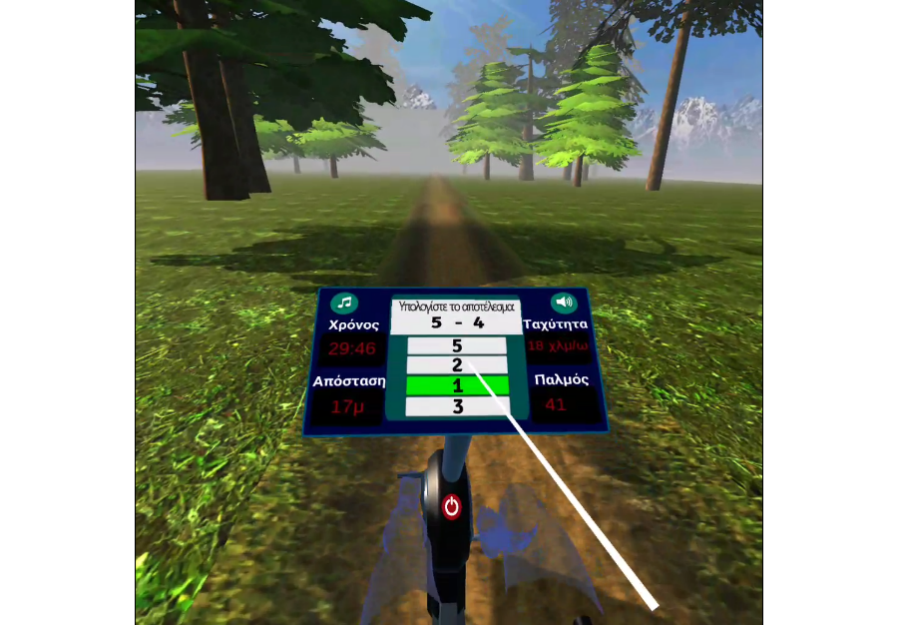
User selects the answer to the math calculation exercise.

At this point, the user can begin cycling on the training bike. During cycling, the user can choose to listen to music from a list of preloaded tracks. The images below (Figure 2) show the math quiz, which asks the user to complete a simple subtraction of 2 single-digit numbers. In this example, as the user answered correctly, the answer is highlighted in green.

**Figure 3:**
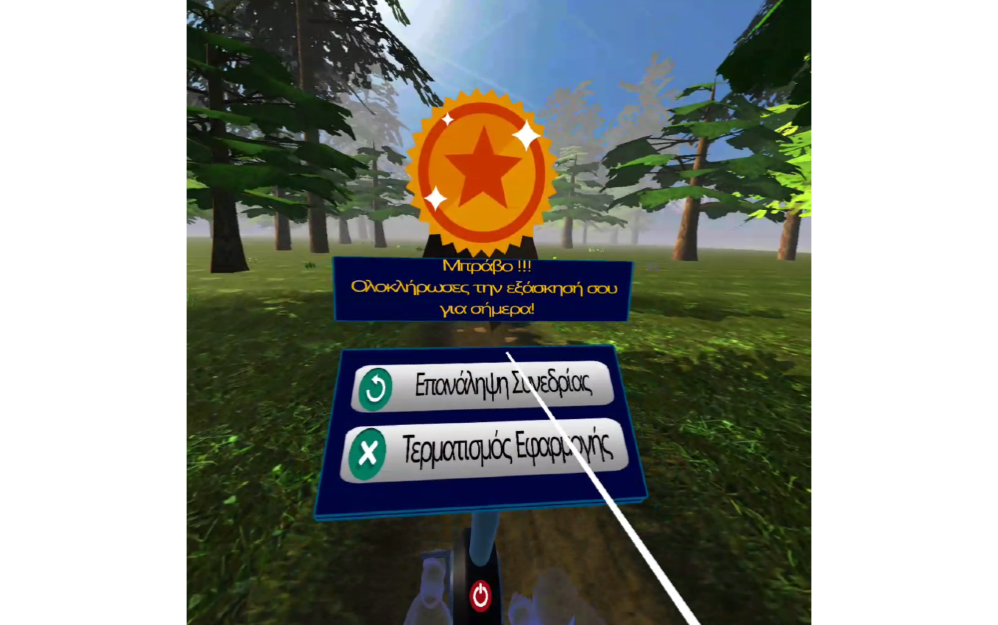
Users’ closing screen after completion of the session.

After completing the cycling session, the user analytics were displayed in front of the bike, identifying correct and incorrect answers from the math quiz. Finally, the user was asked to evaluate their performance and report any difficulties (Figure 3).

It is important to mention that user data were tracked and exported in an analytics file at the end of each session. This file, containing the answers from the math quiz and user-stated preferences, was also saved to the headset.

#### Task and Conditions

Each participant was asked to perform a dual task protocol combining physical and cognitive tasks under 2 different training conditions. The physical aspect of the task involved cycling (on a stationary seated bike) for 15 consecutive minutes at a constant speed of 15 km/h. In every trial, the bike workload was initially self-selected by the user and dynamically adjusted by the supervisor in accordance with the exercise protocol. The cognitive task required participants to complete 20 simple numerical calculations (single-digit additions and subtractions) during cycling. In one condition, participants had to execute the cycling task in the lab environment and were asked by the experimenter to perform the calculations orally. In the other condition, participants were required to execute the cycling task in the virtual environment and were asked to perform the calculations that appeared in the VR app using a remote control.

#### Measures

##### Personal Innovativeness

Personal innovativeness was measured by using 4 items assessing an individual’s general tendency to try out new information technologies [62] adopted from Yusoff et al [63] (eg, I am the kind of person who looks forward to experimenting with new technologies). Responses on a 5-point Likert scale ranged from 1 (strongly disagree) to 5 (strongly agree).

##### Acceptance

Participants’ acceptance of the VRADA app was assessed in terms of 3 factors: perceived enjoyment, attitude, and intended future use. Perceived enjoyment was measured by 6 items that assessed feelings of pleasure while exercising (eg, I really enjoyed exercising in the VR environment) [63]. Responses on a 5-point Likert scale ranged from 1 (strongly disagree) to 5 (strongly agree). Attitudes toward the VRADA app were assessed on the basis of guidelines from planned behavior theory [64] involving 6 bipolar items (eg, pleasant-unpleasant, useful-useless) and scored on a 7-point semantic differential scale. Items assessing intended future use were adapted from a previous study [63] and modified according to the guidelines for assessment of attitudes based on the theory of planned behavior [65]. Three items evaluated the extent to which a person had formulated a conscious plan to exercise in the future in the VR environment (eg, assuming I have access to the system, I intend to use it). Responses on a 5-point Likert scale ranged from 1 (strongly disagree) to 5 (strongly agree).

##### Usability

The system usability scale (SUS) [66] was used to assess subjective components of usability. The SUS is a self-report questionnaire comprising 10 items—5 positive (eg, I think that I would like to use this system frequently) and 5 negative (eg, I found the system unnecessarily complex). Responses were rated on a 5-point Likert scale ranging from 1 (strongly disagree) to 5 (strongly agree).

##### Preference

Preference for the 2 exercise protocols was assessed by 8 questions about exercising in the natural or the virtual environment (eg, Exercise was more pleasant..., the numerical calculations were more fun..., time passed quicker..., when exercising with or without the VR mask). Responses were dichotomous, scoring −1 (against VR) or +1 (in favor of VR).

##### Additional Assessments

A questionnaire and semistructured interviews were conducted to collect additional information immediately following the session. The questionnaire [37] included 9 questions assessing participants’ perceptions of using the Oculus Go headset and controller and the environment in terms of (1) usability—pleasantness (4 items; eg, I felt comfortable using the mask; I enjoyed the park ride); (2) usability—learning, (2 items, eg, It was easy to read the numerical questions); and (3) tolerability (3 items assessing dizziness, boredom, and anxiety). Responses on a 5-point Likert scale ranged from 1 (strongly disagree) to 5 (strongly agree). The semistructured interview [67] collected qualitative information to further explore participants’ subjective perceptions and feelings regarding reasons to use VRADA (eg, Why would you use the VRADA training system?); expectations after the session (2 items; eg, given the opportunity, would you be willing to use this training system regularly?); usability or utilization (5 items; eg, What difficulties did you encounter during the training session?); usability or learning (2 items; eg, Did you have to ask for help to be able to use the system? Where exactly?); usability or pleasantness (2 items; eg, What exactly did you like most and least?); sense of presence or spatial presence (2 items; eg, Did you feel you had control over the environment?); sense of presence or engagement (2 items; eg, Did you get distracted during exercise? By what?), sense of presence or realism (How did you find the environment—realistic or too artificial?); and tolerability (2 items; eg, Did you feel bad during exercise? When and where exactly?). A detailed interview guide can be found in Multimedia Appendix 3.

#### Procedure

As volunteers, participants signed consent forms after being provided with an information sheet describing the study requirements and confirming their right to withdraw from the study at any time. In the preparatory phase of the experimental session, participants were told about the procedure and were encouraged to ask questions. They were then equipped with a VR mask and remote control and were allowed some time to familiarize themselves with this equipment. They were then brought to the stationary bike to make seat adjustments and to become familiar with the task, which involved cycling for 2 minutes. Two training conditions were implemented to complete this preparatory phase. The order of the conditions was counterbalanced, with a 10-minute break between the 2 conditions. Finally, after a 5-minute rest, the participants completed the questionnaire and discussed the interview questions with the experimenter. The entire procedure took approximately 60 minutes to complete.

#### Data Analysis

Quantitative data were analyzed using SPSS Statistics version 21 (IBM Corporation). Summary statistics were calculated for demographic characteristics, and correlations among all examined variables were assessed using the Pearson coefficient. Exercise protocol preference was examined using a single-sample two-tailed *t* test, using *P*<.05 (two-sided) to determine statistical significance. The qualitative interview data were analyzed using thematic analysis [68], which can offer rich insights into attitudes and beliefs by identifying patterns of ideas or responses. As the discussion topics were based on relevant previous studies, the main themes were predetermined (deductive approach). Second-order themes were analyzed using an inductive approach, allowing the data to determine subthemes.

### Ethics Approval and Consent to Participate

The institution’s ethics committee granted permission for these studies (approval number: 1557, October 2, 2019). The confidentiality of private personal and health information will be ensured in line with regulations (European Union, EU) 2016/679 (General Data Protection Regulation). Participants were briefed verbally, face-to-face, and provided written information, including the consent form. Where necessary, participants were provided with additional information about the study.

## Results

### Formative Study VR1

Table 1 summarizes the participants’ answers regarding their past experiences of physical activity, along with their preferences and expectations for a VR system combining physical and cognitive training.

The results were presented to the research group, comprising providers of patients’ physical and cognitive training, their neuropsychiatrist (extensive clinical and research experience in dementia), exercise psychologists (specialists in exercise motivation), a biomedical engineer, and a computer scientist specializing in computer graphics and extended reality. On the basis of their expertise and the input from patients with MCI, the group made collaborative decisions on the design and content of the first VRADA prototype.

**Table 1:** Experiences, preferences, and expectations of patients with mild cognitive impairment regarding a virtual reality training environment.

Topic				Values
**Current physical activity**				
	**Type of exercise, n (%)**			
		Full body exercises	15 (75)	
		Neck and shoulder exercises	3 (15)	
		Walking	2 (10)	
	Frequency of exercise (times/week), mean (SD)			2.70 (1.55)
	Duration of exercise (min/training), mean (SD)			53.75 (11.57)
**Ideal exercise environment**				
	**Scenery, n (%)**			
		Forest or park	11 (55)	
		Seaside	6 (30)	
		Town	3 (15)	
	**Season, n (%)**			
		Spring	11 (52)	
		Autumn	6 (30)	
		Winter	2 (12)	
		Summer	1 (6)	
	**Time of day, n (%)**			
		Morning	18 (90)	
		Night	2 (10)	
	**Social environment** **(** **exercise with other), n (%)**			
		Yes	18 (90)	
		No	2 (10)	
	**Natural environment, n (%)**			
		Sounds of nature	6 (30)	
		Birdsong	7 (35)	
		Waves splashing	1 (5)	
		Other	6 (30)	
	**Music, n (%)**			
		Soft classic music	9 (45)	
		Traditional Greek music	5 (25)	
		No music	6 (30)	
**Feedback during exercise**				
	**Time, speed, distance, and heart rate, n (%)**			
		Yes	18 (90)	
		No	2 (10)	
	**Feedback presentation, n (%)**			
		Monitor	10 (50)	
		From training provider	10 (50)	
**Type of bike**				
	**Familiar with bike, n (%)**			
		Yes	16 (80)	
		No	4 (20)	
	**Balance on stationary bike, n (%)**			
		Yes	16 (80)	
		No	4 (20)	
	**Type of bike, n (%)**			
		Seated bike	16 (80)	
		Upright bike	4 (20)	
**Cognitive exercises**				
	**Number calculations, n (%)**			
		Yes	15 (73)	
		No	5 (27)	
	**First letter task, n (%)**			
		Yes	17 (85)	
		No	3 (15)	
	**Anagrammatical task, n (%)**			
		Yes	10 (50)	
		No	10 (50)	
	**Synonyms-antonyms task, n (%)**			
		Yes	14 (70)	
		No	6 (30)	
	**Missing words task, n (%)**			
		Yes	15 (75)	
		No	5 (25)	
	**Create sentences task, n (%)**			
		Yes	18 (90)	
		No	2 (10)	

#### Building the VRADA Training System

On the basis of information from participant interviews, health professionals from Alzheimer Hellas Day Care Centers, and experts from the exercise psychology research group, the ORAMA-VR team prepared the first prototype, and the biomechanical solutions engineering team incorporated the VRADA software into the bike (Toorx, Chrono Line, BRX R 300). The development procedure was based on continuous testing feedback; that is, each software version was user-tested to inform further development of the prototype. As we felt this might prove burdensome for the intended end users (ie, older people with MCI), university students tested the prototype and provided the necessary feedback.

During this period, a bridge device was used to connect the VRADA software to the bike. Other tasks performed during this period included continuous improvement of the VRADA visual environment, introduction and thorough testing of cognitive exercises, construction of data storage and extraction mechanisms, and adjustments to regulation of real and virtual speeds. Throughout this continuous testing period, student users were asked open questions about specific aspects of the development process—for example, alignment of bike pedaling speed with the VR biking experience. Similarly, when adding cognitive exercises to the VR environment, feedback was collected from a series of trials to improve the design and ensure smooth alignment with the VR biking experience.

#### System Architecture

The VRADA app was built on top of the ORamaVR MAGES platform [69,70], using the latter’s training and interaction mechanics. The MAGES platform is fully customizable and supports educational VR simulations with minimal adaptation. This is accomplished by prototyping the learning pipeline into structured, independent, and reusable segments, which are used to generate more complex behaviors. The architecture supports all current and forthcoming VR head-mounted displays and standard 3-dimensional content generation. The MAGES platform includes the following novel features [70]:

- *Multiplayer with geometric algebra interpolation: custom low-bandwidth and high visual fidelity collaborative modules*. Our geometric algebra framework enables 4 times the improvement in reduced data network transfer and lower processor usage.
- *Analytics based on machine learning agents with recommendations*: We used medical experts to train our machine learning agent and constructed a unique trainee profile to make real time suggestions to users according to their level of experience. Our supervised machine learning model is capable of understanding the validity of each action and deciding whether to offer assistance in the form of additional audio-visual guidance.
- *Geometric algebra deformable animation, cutting, and tearing*: The use of quaternions and dual quaternions yielded fast results, with no interpolation problems or other geometric artifacts. Our engine also performs animations with fewer intermediate keyframes, thereby reducing the bandwidth.
- *Editor in VR*: This module allows non-VR experts to develop new modules or scenarios or to modify existing ones in a coding-free environment.
- *Semantically annotated bodies*: The MAGES core includes an advanced mathematical algorithm for physics-based visual techniques that can generate a virtual representation of the body, which is essential for VR physical training.

#### Virtual Environment

**Figure 4:**
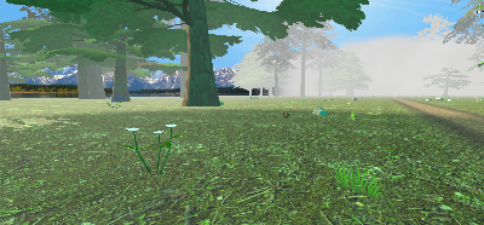
The dynamically generated forest.

We designed a forest path as a scenery for a relaxing and enjoyable virtual environment. The forest is dynamically generated as the user cycles along the path (Figure 4). We implemented this mechanism to optimize performance, as the app is deployed on a mobile VR headset, and its performance is limited by the onboard graphics chip. In addition, we populated the forest with animals that the user must remember for the purposes of the memory game at the end of the session.

#### Head-Mounted Display

**Figure 5:**
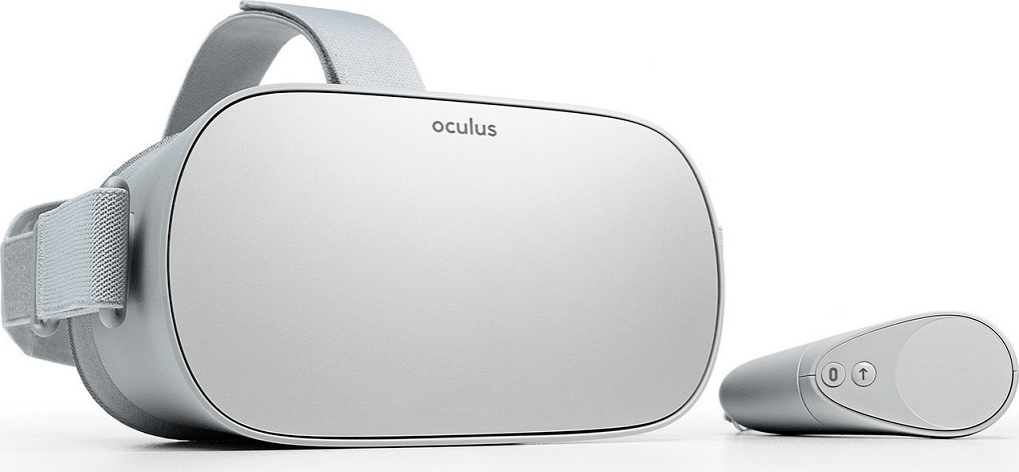
The Oculus Go Head-Mounted display.

The VRADA app uses Oculus Go as the main VR head-mounted display (Figure 5). Oculus Go is a 3DOF (degrees of freedom) headset with a single 3DOF controller. As a standalone untethered headset, it does not need a desktop connection, and the absence of cables makes the device mobile and ideal for use while exercising.

### Feasibility Studies VR2a and VR2b

When the development of the first VRADA prototype reached a satisfactory level, we conducted an early feasibility trial, focusing on acceptability, usability, and tolerability, first with university students and then with the intended end users (Alzheimer Hellas for Alzheimer Association). The efficacy of the final version of the system will be tested in a future RCT (VR3).

Tables 2 and 3 presents the means, SD, Cronbach α, and Pearson correlations for Studies VR2a and VR2b. All scales from both studies exhibited high internal consistency (Cronbach α .71 and .89), with the exception of the SUS, which returned lower (.67 and .68 for VR2a and VR2b, respectively) but still acceptable internal consistency [71].

**Table 2:** Descriptive statistics for study VR2a, including Cronbach α and Pearson correlations.

Variables			Study VR2a															Mean (SD)				Cronbach α	
			PI^a^	PE^b^		ITU^c^		ATT^d^		SUS^e^	PREF^f^	UP^g^		UL^h^									
**PI**																			3.68 (0.65)				.76
	*r*	—^i^																					
	*P* value	—																					
**PE**																			4.22 (0.61)				.88
	*r*	0.14			—																		
	*P* value	.45			—																		
**ITU**																			3.91 (0.95)				.89
	*r*	0.18			0.78		—																
	*P* value	.34			<.001		—																
**ATT**																			6.12 (0.78)				.79
	*r*	−0.00			0.67		0.53		—														
	*P* value	.96			<.001		<.001		—														
**SUS**																			82.66 (9.00)				.67
	*r*	0.13			0.48		0.24		0.47	—													
	*P* value	.49			<.001		.19		<.001	—													
**PREF**																			0.65 (0.41)				.71
	*r*	0.00			0.28		0.44		0.24	0.10	—												
	*P* value	.96			.13		.01		.19	.58	—												
**UP**																			4.33 (0.58)				.58
	*r*	0.31			0.54		0.28		0.35	0.52	−0.04		—										
	*P* value	.08			<.001		.12		.05	<.001	.83		—										
**UL**																			4.40 (0.66)				.59
	*r*	0.21			0.46		0.20		0.43	0.59	0.12		0.43		—								
	*P* value	.25			.01		.28		.01	<.001	.51		.01		—								
**TOL^j^**																			3.94 (0.89)				.61
	*r*	0.19			0.41		0.42		0.27	0.60	0.51		0.56		0.34								
	*P* value	.30			.02		.02		.14	<.001	<.001		<.001		<.001								

^a^PI: personal innovativeness.

^b^PE: perceived enjoyment.

^c^ITU: intention to use.

^d^ATT: attitudes.

^e^SUS: system usability scale.

^f^PREF: preferences.

^g^UP: usability-pleasantness.

^h^UL: usability-learning.

^i^Not applicable.

^j^TOL: tolerability.

**Table 3:** Descriptive statistics for study VR2b, including Cronbach α and Pearson correlations.

Variables			Study VR2b																	Mean (SD)			Cronbach α
			PI^a^		PE^b^		ITU^c^		ATT^d^		SUS^e^		PREF^f^		UP^g^		UL^h^						
**PI**																				3.86 (0.82)			.87
	*r*	—^i^																					
	*P* value	—																					
**PE**																				4.43 (0.57)			.81
	*r*	0.36		—																			
	*P* value	.06		—																			
**ITU**																				4.19 (0.78)			.95
	*r*	0.44		0.82		—																	
	*P* value	.02		< .001		—																	
**ATT**																				6.17 (1.00)			.74
	*r*	0.05		0.47		0.34		—															
	*P* value	.79		.01		.07		—															
**SUS**																				77.96 (13.40)			.68
	*r*	0.48		0.68		0.70		0.43		—													
	*P* value	.01		< .001		< .001		.02		—													
**PREF**																				0.72 (0.51)			.89
	*r*	0.06		0.23		0.22		0.37		0.10		—											
	*P* value	.73		.24		.26		.05		.61		—											
**UP**																				4.77 (0.52)			.73
	*r*	0.02		0.51		0.42		0.38		0.37		0.52		—									
	*P* value	.89		.006		.02		.05		.05		< .001		—									
**UL**																				4.53 (0.67)			.94
	*r*	0.00		0.27		0.13		0.43		0.28		0.41		0.59		—							
	*P* value	.99		.16		.51		.02		.15		.03		< .001		—							
**TOL^j^**																				4.26 (0.96)			.89
	*r*	0.13		0.26		0.36		0.48		0.47		0.38		0.53		0.33							
	*P* value	.50		.17		.06		.01		.01		.05		.01		.09							

^a^PI: personal innovativeness.

^b^PE: perceived enjoyment.

^c^ITU: intention to use.

^d^ATT: attitudes.

^e^SUS: system usability scale.

^f^PREF: preferences.

^g^UP: usability-pleasantness.

^h^UL: usability-learning.

^i^Not applicable.

^j^TOL: tolerability.

#### Personal Innovativeness, Acceptability, and SUS

In VR2a, students scored moderately to high on personal innovativeness and intended future use and high on attitudes toward VR exercise and enjoyment. The score for usability (82.66/100) was well above the acceptability threshold (75/100).

In study VR2b, patients with MCI scored moderately to high on personal innovativeness and high on enjoyment, attitude to VR exercise, and intended future use. The usability score (77.96) was also above the acceptability threshold.

#### Preferences

A single-sample *t* test was performed to determine whether there was a statistically significant preference for either of the 2 conditions, coding the normal environment as −1 and the VR environment as +1, with the test value set at 0. For study VR2a, the analysis indicated a significant preference for the VR condition (mean 0.66, SD 0.41; t_29_=8.74; *P*<.001). Similarly, for study VR2b, the analysis indicated a significant preference for the VR condition (mean 0.72, SD 0.51; t_26_=7.36; *P*<.001).

#### Pearson Correlations

Pearson correlations between all variables were calculated for the 2 studies (Tables 2 and 3). In study VR2a, innovativeness was unrelated to any of the other variables; acceptance variables were strongly interrelated and moderately related to usability. Finally, preference was most strongly related to intention. In study VR2b, innovativeness was moderately related to usability and to 2 acceptance variables (enjoyment and intention). Acceptance variables were positively interrelated and strongly related to usability. Finally, preference was most strongly related to attitude, but the relationship was not statistically significant.

#### Evaluation of Headset, Controller, and VRADA Environment

In study VR2a, students’ scores were high for the VR gear, VRADA environment, preference, and usability and moderate to high for acceptance variables and innovativeness. In study VR2b, MCI patients’ scores were high for the VR gear, VRADA environment, preference, and usability and moderate to high for acceptance variables and innovativeness.

The semistructured interview data regarding usability, sense of presence, tolerability, and expectations are summarized in Table 4. In both groups, most participants reported a preference for the VRADA training system compared with standard care training. Comments in relation to most dimensions of usability were also very similar in both groups, but the MCI patient group reported needing more help when learning how to use the VR equipment. The 2 groups differed in relation to perceived feeling of presence, but engagement and realism (as dimensions of sense of presence) exhibited the same direction. Surprisingly, tolerability was higher among the MCI patients, as was the intended future use of the system.

**Table 4:** Summary of interview data: students and patients with mild cognitive impairment.

Main theme	Subthemes	
	Study VR^a^2a (students)	Study VR2b (patients with MCI^b^)
Reasons to use VRADA^c^	Because: ...“VRADA is more pleasant and interesting” (51%) ...“time passes faster” (31%) ...“it is less boring” (10%) ...“it is less tedious” (8%)	Because: ...“VRADA is more pleasant, beautiful, and interesting” (56%) ...“time passes faster” (28%) ...“it is like escaping from reality” (12%) ...“it improves visibility” (4%)
Expectations	Future personal use of the system: Yes: 50%; So-so: 33%; No: 17% System is useful for other populations (young 35%, obese 7%, disabilities 39%, older people 19%)	Future personal use of the system: Yes: 81%; So-so: 12%; No: 7% System is useful for other populations (young 18%, everybody 23%, disabilities 41%, people who like to explore nature 18%)
Usability	Utilization: General difficulties (no difficulties 64%, VR controller 20%, dizziness 8%, sweat 8%) Technical problems (none 79%, connectivity 21%) VR controller use (ΟΚ 75%, uncomfortable handle 17%, sensitivity 8%) VR mask use (OK 89%, blur 11%) Learning to use: Need for extra help: No 100% Need more time to understand the system: No 100% Pleasantness: Most enjoyable parts (environment 68%, music 32%) Least enjoyable parts (repeated virtual parts 48%, graphics 37%, music 15%) Feel uncomfortable: No 83%, dizziness 17%	Utilization: General difficulties (no difficulties 63%, VR controller 23%, speed 14%) Technical problems (none 87%, connectivity 13%) VR controller use (ΟΚ 50%, control and sensitivity 50%) VR mask use (OK 95%, dysphoria 5%) Learning to use: Need for extra help: No 78%, Yes (how to start) 13%; Yes (how to use VR controller) 9% Need more time to understand the system: (No 88%, Yes 12%) Pleasantness: Most enjoyable parts (environment 92%, animals 8%) Least enjoyable parts (repeated virtual parts 75%, VR controller 13%, graphics 12%) Feel uncomfortable: No 95%, VR mask 5%
Sense of presence	Spatial presence: Feeling of presence: Yes 23%, So-so 67%, No 10% Control of the system: Yes 90%, So-so 10% Engagement: Duration of experience (prefer more 59%, prefer less 41%); distraction of attention: No 68%, cognitive exercises 32% Realism: Realistic or artificial virtual environment (realistic 3%, so-so 20%, artificial 77%)	Spatial presence: Feeling of presence: Yes 77%, So-so 12%, No 11%) Control of the system: Yes 88%, So-so 4%, No 8% Engagement: Duration of experience (prefer more 53%, good 47%), distraction of attention: No 76%, VR controller 12%, cognitive exercises 12% Realism: Realistic or artificial virtual environment (realistic 34%, artificial 66%)
Tolerability	Feel bad during training: No 85%, Yes 15% Feel nausea, dizziness, or other physical symptoms: No 62%, Yes 38%	Feel bad during training (No 93%, Yes 7%) Feel nausea, dizziness, or other physical symptoms: No 93%, Yes 7%)

^a^VR: virtual reality.

^b^MCI: mild cognitive impairment.

^c^VRADA: VR Exercise App for Dementia and Alzheimer’s Patients.

## Discussion

### Principal Findings

The development of the VRADA training system followed the latest proposed recommendations and strategies [56,57] for VR therapeutic and health apps. In line with these guidelines, we focused on the development of the content, VR environment, and system architecture (VR1). In collaboration with patients and health care providers and on the basis of the principles of human-centered design and continuous testing, VRADA incorporated dual task cognitive and physical training for older patients with MCI in a user-friendly VR environment.

The quantitative and qualitative data (VR2) regarding the acceptability, usability, and tolerability of VRADA as a training system are encouraging. As evidenced by the scores across all acceptability dimensions, the system was well accepted by both test groups. Acceptability is a key issue for innovative technology in the treatment schemes of neurodegenerative diseases, especially among the older population [37,38,72,73]. Scores for perceived enjoyment, attitude, and intention to use were well above average, with end users scoring higher than the students’ group on all dimensions. The MCI patient interview comments were also very encouraging regarding the future use of VRADA, as they found the system more pleasant and interesting. This positive attitude toward VR among older people aligns with previous research. One systematic review [74] reported evidence that technology is a well-accepted method to provide engaging exercise opportunities to older people, and the high adherence rates can be explained largely by the high reported levels of enjoyment they experience when using these programs.

The SUS results were also very encouraging, indicating that the system exhibited good usability. Developing a usable, immersive VR system for older people at risk of cognitive decline requires careful consideration, and our design followed the latest recommendations [75] for developing similar VR platforms. We involved an interdisciplinary group of experts from the early stages of development and tried to meet basic psychological needs [76] by promoting user autonomy by providing choices. We also addressed their need to feel competent by providing encouragement, feedback, and an easy-to-use system that contributed to enjoyment and satisfaction. As almost half of our older participants were relatively unfamiliar with novel technology use, we provided a short initial training element to familiarize them with VR. However, the end users’ comments suggest a need to provide more detailed instructions on using the system; in particular, some additional time might be needed for them to become familiar with the controller.

Simulator sickness, which can be attributed to postural instability or sensory conﬂict, is often a concern when older people use immersive VR [77]. However, most participants from both groups (93%) tolerated the VRADA training system very well, with no adverse effects (eg, nausea, dizziness, and anxiety) among the patients with MCI, who perceived it as an enjoyable experience. Finally, the correlation results show that personal innovativeness was unrelated to any of the other variables, indicating that the system may be attractive and interesting even for less innovation–seeking users, at least within this sample.

### Strengths and Limitations

The VRADA training system is among the first to attempt to transfer standard care, nonpharmacological, cognitive, and physical training as a simultaneous dual task MCI treatment scheme to a virtual environment. According to the literature [78], direct and indirect interventions targeting cognitive-motor interference have shown promise as a means of improving MCI in individuals with neurodegenerative diseases. Although similar previous studies have reported encouraging results [37,38], the dual task training program was sequential rather than simultaneous. Moreover, VR technology training environments such as VRADA provide flexibility in clinical settings because they can be tailored to individual needs and facilitate training in settings that are either impossible or unsafe in the real world. VRADA may also help to increase older people’s autonomy and has the potential to reduce the workload of health care professionals dealing with patients with MCI. Finally, VRADA can be combined with other techniques, such as functional magnetic resonance imaging, to track brain functionality in the VR environment. This can provide further valuable insights into the effects of VR [79].

Despite these positive results regarding acceptability, usability, and tolerability, this study has some limitations. As the VRADA training system was only tested in a single session, we cannot conclude that this training would remain interesting and enjoyable after repeated longer sessions, and future studies should explore this issue. It is also important to emphasize that this study was designed to test the feasibility of the VRADA training system but not its efficacy. We intend to test efficacy in the near future in an RCT that will examine whether regular training using the VRADA system results in improved physical, cognitive, and quality of life outcomes.

### Conclusions

This study addressed the design of a user-friendly, acceptable, and tolerable immersive VR system for dual task physical and cognitive skills training. Both students and older people with MCI symptoms reported high levels of acceptability, usability, and tolerability when using the VRADA training system, confirming its potential (subject to RCT efficacy validation) as a tool to promote physical and cognitive health among patients with MCI.

## Appendix

Multimedia Appendix 1 Baseline demographic characteristics: studies VR2a and VR2b.

Multimedia Appendix 2 Video of the virtual reality Exercise App for Dementia and Alzheimer’s Patients training environment.

Multimedia Appendix 3 Interview guide.

## Abbreviations

AD: Alzheimer disease
DOF: degrees of freedom
EU: European Union
MCI: mild cognitive impairment
RCT: randomized controlled trial
SUS: system usability scale
VR: virtual reality
VRADA: VR Exercise App for Dementia and Alzheimer’s Patients
WHO: World Health Organization
