# Supplementary material for: Α Virtual Reality App for Physical and Cognitive Training of Older People With Mild Cognitive Impairment: Mixed Methods Feasibility Study
Source: JMIR Serious Games. 2021 Mar 24;9(1):e24170. doi: 10.2196/24170 (PMC8294639; doi:10.2196/24170)
Supplement: Multimedia Appendix 1 [file games_v9i1e24170_app1.docx]

**Multimedia appendix 1.** Baseline demographic characteristics: studies VR2a and VR2b.

| Demographics | | | | Values |
| --- | --- | --- | --- | --- |
| **Study VR2a** | | | | |
|  | **Educational level, %** | | | |
|  |  | Primary | | 0 (%) |
|  |  | Secondary | | 0 (%) |
|  |  | Higher | | 100 (%) |
|  | **Physical activity, %** | | | |
|  |  | **Times per week** | | |
|  |  |  | 1 | 4 (%) |
|  |  |  | 2 | 14 (%) |
|  |  |  | 3 | 38 (%) |
|  |  |  | 4 | 11 (%) |
|  |  |  | 5 | 4 (%) |
|  |  |  | 6 | 25 (%) |
|  |  |  | 7 | 4 (%) |
|  |  | **Time per training (min)** | | |
|  |  |  | 10 | 4 (%) |
|  |  |  | 20 | 0 (%) |
|  |  |  | 30 | 11 (%) |
|  |  |  | 45 | 7 (%) |
|  |  |  | 60 | 21 (%) |
|  |  |  | 90 | 39 (%) |
|  |  |  | 120 | 18 (%) |
|  | **Technology use** | | | |
|  |  | **Phone use, %** | | |
|  |  |  | Never | 0 (%) |
|  |  |  | 1-2 hours | 20 (%) |
|  |  |  | 3-4 hours | 53 (%) |
|  |  |  | 5-6 hours | 10 (%) |
|  |  |  | >7 hours | 17 (%) |
|  |  | **Computer use, %** | | |
|  |  |  | Never | 20 (%) |
|  |  |  | 1-2 hours | 53 (%) |
|  |  |  | 3-4 hours | 17 (%) |
|  |  |  | 5-6 hours | 7 (%) |
|  |  |  | >7 hours | 3 (%) |
|  |  | **Gaming use, %** | | |
|  |  |  | Never | 73 (%) |
|  |  |  | 1-2 hours | 23 (%) |
|  |  |  | 3-4 hours | 4 (%) |
|  |  |  | 5-6 hours | 0 (%) |
|  |  |  | >7 hours | 0 (%) |
| **Study VR2b** | | | | |
|  | **Educational level, %** | | | |
|  |  | Primary | | 31 (%) |
|  |  | Secondary | | 42 (%) |
|  |  | Higher | | 27 (%) |
|  | **Physical activity, %** | | | |
|  |  | **Times per week** | | |
|  |  |  | 1 | 12 (%) |
|  |  |  | 2 | 21 (%) |
|  |  |  | 3 | 17 (%) |
|  |  |  | 4 | 25 (%) |
|  |  |  | 5 | 21 (%) |
|  |  |  | 6 | 4 (%) |
|  |  |  | 7 | 0 (%) |
|  |  | **Time per training (min)** | | |
|  |  |  | 10 | 0 (%) |
|  |  |  | 20 | 0 (%) |
|  |  |  | 30 | 0 (%) |
|  |  |  | 45 | 25 (%) |
|  |  |  | 60 | 71 (%) |
|  |  |  | 90 | 4 (%) |
|  |  |  | 120 | 0 (%) |
|  | **Technology use** | | | |
|  |  | **Phone use, %** | | |
|  |  |  | Never | 4 (%) |
|  |  |  | 1-2 hours | 88 (%) |
|  |  |  | 3-4 hours | 4 (%) |
|  |  |  | 5-6 hours | 0 (%) |
|  |  |  | >7 hours | 4 (%) |
|  |  | **Computer use, %** | | |
|  |  |  | Never | 44 (%) |
|  |  |  | 1-2 hours | 30 (%) |
|  |  |  | 3-4 hours | 18 (%) |
|  |  |  | 5-6 hours | 4 (%) |
|  |  |  | >7 hours | 4 (%) |
|  |  | **Gaming use, %** | | |
|  |  |  | Never | 55 (%) |
|  |  |  | 1-2 hours | 30 (%) |
|  |  |  | 3-4 hours | 7 (%) |
|  |  |  | 5-6 hours | 4 (%) |
|  |  |  | >7 hours | 4 (%) |
